# One Health Probiotics as Biocontrol Agents: One Health Tomato Probiotics

**DOI:** 10.3390/plants11101334

**Published:** 2022-05-18

**Authors:** Natalya Harutyunyan, Almagul Kushugulova, Narine Hovhannisyan, Astghik Pepoyan

**Affiliations:** 1 Food Safety and Biotechnology Department, Armenian National Agrarian University, 74 Teryan St., Yerevan 0009, Armenia; natalya.harutyunyan@list.ru; 2 Laboratory of Human Microbiome and Longevity, Center for Life Sciences, National Laboratory Astana, Nazarbayev University, 53 Kabanbay Batyr Ave., Nur-Sultan 010000, Kazakhstan; akushugulova@nu.edu.kz; 3 Plant Origin Raw Material Processing Technology Department, Armenian National Agrarian University, 74 Teryan St., Yerevan 0009, Armenia; narinehovhannisyan1984@mail.ru

**Keywords:** tomato, rhizosphere bacteria, tomato processing, *Lactiplantibacillus plantarum*, “One Health” probiotic

## Abstract

Tomato (*Lycopersicon esculentum*) is one of the most popular and valuable vegetables in the world. The most common products of its industrial processing in the food industry are juice, tomato paste, various sauces, canned or sun-dried fruits and powdered products. Tomato fruits are susceptible to bacterial diseases, and bacterial contamination can be a risk factor for the safety of processed tomato products. Developments in bioinformatics allow researchers to discuss target probiotic strains from an existing large number of probiotic strains for any link in the soil–plant–animal-human chain. Based on the literature and knowledge on the “One Health” concept, this study relates to the suggestion of a new term for probiotics: “One Health probiotics”, beneficial for the unity of people, animals, and the environment. Strains of *Lactiplantibacillus plantarum*, having an ability to ferment a broad spectrum of plant carbohydrates, probiotic effects in human, and animal health, as well as being found in dairy products, vegetables, sauerkraut, pickles, some cheeses, fermented sausages, fish products, and rhizospheric soil, might be suggested as one of the probable candidates for “One Health” probiotics (also, for “One Health—tomato” probiotics) for the utilization in agriculture, food processing, and healthcare.

## 1. Introduction

Tomato (*Lycopersicon esculentum*) is one of the leaders in the classification of useful products. It is also one of the most popular and valuable vegetables in the world [1]. It contains many useful compounds, such as ascorbic acid [2], lycopene, β-carotene, anthocyanin, and others [3,4]. The content of trace elements and the above-mentioned compounds in tomatoes also create prerequisites for their use as components in various diets, and they can be used to reduce the risk factors of many diseases (cancer, osteoporosis, cardiovascular diseases) [1,5]. Wild tomato species and varieties have a rich potential for genetic diversity and greatly contribute to the selection of new, valuable genotypes with high productivity and an ability to adapt to stress. Despite researchers’ ongoing interest in transgenic crops [6,7], genetically modified crops, including tomatoes, can cause unpredictable environmental problems [8]. Currently, the selection of tomato varieties with valuable properties is a primary task for the researchers. Analysis in the field of breeding allows us to conclude that the genomic potential of tomato species is not fully used and there is a possibility of a wide choice of varieties. During recent years, tomato species have been supplemented with new, high-yielding varieties and hybrids, which are not only more resistant to diseases and pests, but also contain more vitamin C, total sugar, dry matter and acidity.

The most common products from the industrial processing of tomato fruit in the food industry are juice, tomato paste, various sauces, canned or sun-dried fruits and powdered products. Methods for obtaining various concentrates containing biologically active compounds (including carotenoids and lycopene) from tomatoes, as well as from the byproducts of their processing (pulp, skin and seeds), are well known [9]. Tomatoes are also a subject of interest for the cosmetic and perfumery industries when developing organic cosmetics line [10]. If a cosmetic contains individual components of tomatoes (lycopene, quercetin, salicylic acid, vitamin C, β-carotene), it will have a protective effect on skin from ultraviolet radiation and will slow down the aging of the skin by reducing the number of free radicals [11]. Due to the presence of sterols and vitamin E, tomato seed oil allows us to restore the protective barrier of the skin, thereby increasing its overall level of moisturization. Salicylic acid is effective in the treatment of inflammatory processes of the skin (acne). It has an antibacterial, keratolytic effect. In addition, lycopene stimulates the production of antioxidant enzymes that prevent the development of inflammation [12]. However, tomato fruits are susceptible to bacterial diseases, the intensity of the development of which depends both on the characteristics of the processing of the plant and on their general condition. In addition, bacterial contamination is a risk factor for the safety of processed tomato products, such as tomato juice and tomato paste.

One Health is the concept of the interconnection of human, animal and environmental wellbeing [13,14]. The concept focuses on interactions between humans and their environment as a trigger for health and disease mainly through the cycling of environmental microbial communities [15]. Here, we hope to draw the attention of researchers to tomato-probiotics/“One Health” probiotics to protect both fruits and tomato products from bacterial infections.

## 2. Soil—Tomato Rhizosphere Bacteria

Soil has a significant impact on the promotion of plant growth and productivity, mainly through plant–rhizosphere microbiome interaction [16,17]. Rhizobacteria, having a direct effect on the modulation of phytohormone levels, ammonia production and phosphate solubilization, may also have antibiotic, siderophore and hydrogen cyanide production effects [18]. Overall, the plant growth-promoting bacteria and mycorrhizal fungi participate in nutrients, mobilizing and stimulating growth and increasing the yield of plants. According to Lee and coauthors, changes in rhizosphere soil microbiota are revealed in association with the healthy or diseased state of the rhizosphere [19]. In addition, host genetics affect human [20,21], animal [22] and plant microbiomes [23,24]. The tomato genotype influences the potential functions of soil bacterial communities. In general, wild tomatoes differ from modern cultivars and tomato landraces [25]. The subgroups of *Rhizobiales*, *Xanthomonadales*, *Burkholderiales*, *Nitrosomona-dales*, *Myxococcales*, *Sphingobacteriales*, *Cytophagales* and *Acidobacteria* from the *Proteobacteria*, *Bacteroidetes* and *Acidobacteriaare* are the dominant tomato rhizosphere bacteria [26]. The strains *Pseudomonas fluorescens*, *Bacillus sp.*, *Azotobacter*, *Serratia*, and *Micromonospora* are involved in tomato disease management and tomato growth promotion [27]. However, the bacteria from the plant rhizosphere microbiome might also compete with other soil rhizobia—the ‘’rhizobial competition problem’’ [28]—and compete with plants for nutrients, or they might act as soilborne plant pathogens [29,30].

Different methods, including spectral [31] and molecular: classical and nested, multiplex, quantitative, bio- and magnetic-capture hybridization polymerase chain reaction, as well as amplification and sequencing methods/tools [32] are currently used for the early detection of bacterial and fungal plant diseases. The main treatment methods for most tomato diseases, for example anthracnose (causative agent: *Colletotrichum* spp.), bacterial canker (causative agent: *Clavibacter michiganensis* subsp. *michiganensis*), bacterial speck (causative agent: * Pseudomonas syringae pv. tomato*), bacterial spot (causative agent: *Xanthomonas* spp.), fusarium wilt disease (causative agent: *Fusarium oxysporum f.* sp. *lycopersici*), bacterial wilt (causative agent: *Ralstonia solanacearum*) and others are fungicides, which might face complications in association with bacterial resistance, which is more related to bacterial spot than to bacterial speck because of the absence of any important problem in the Pto gene. In the case of bacterial canker, two genes for resistance have identified the need for additional investigations on bacterial resistance (http://www.omafra.gov.on.ca/english/crops/facts/05-069.htm) (accessed on 11 May 2020). To avoid major bacterial diseases and to neutralize causative bacteria as reservoirs of diseases, it is also necessary to protect weeds in/around the field (http://www.omafra.gov.on.ca/english/crops/facts/05-069.htm) (accessed on 11 May 2020). Researchers are looking for low-toxicity, high-selectivity, high-activity fungicides to limit the use of several fungicides because of the toxicity of their residues and/or the resistance of plant pathogens to these fungicides [33]. Some of the technologies being examined are: bacteriophages, systemic acquired resistance, bacteriocins and microbial control agents. In comparison with synthetic or chemical fungicides, the “natural” ones (bio fungicides from natural sources), mostly with a plant or microbial origin, are preferable and are widely used as agricultural bioweapons [34]. The investigations of Chanthini and coauthors on the antifungal activity of bacterial cultures against *Alternaria solani* have shown that the spraying bacterial cultures on diseased tomato plants might reduce the severity of blight disease in tomato from 86% to 5.33% [35]. Other studies on the selection of bacterial candidates that are capable of not only preventing the growth of plant pathogens, such as *Botrytis* spp., *Colletotrichum* spp., *Phytophthora* spp. and *Verticillium* spp., but that also are metabolically efficient, revealed effective strains from the genera *Arthrobacter*, *Bacillus*, *Pseudomonas* and *Rhodococcus*, which are also resistant to chemical stress [36].

At the same time, it is known that the applications of bacterial cultures are used for the preservation of tomato products. In vitro screening of 55 strains by antagonistic activity against crop pathogens (*Pseudomonas syringae* pv. *Actinidiae* from the kiwifruit, *Xanthomonas arboricola* pv. *Pruni* from the prunus and *Xanthomonas fragariae* from the strawberry) revealed high activity of the strains *Lactiplantibacillus plantarum* (formerly *Lactobacillus plantarum*) CC100, PM411 and TC92, and of *Leuconostoc mesenteroides* CM160 and CM209 against pathogens [37].

Biotechnological processes related to bacteria make it possible to obtain many valuable chemical compounds. For example, bacterial metabolites may be a potential source of bioactive molecules, such as tripyrrolic, red-colored prodiginines, which not only have antimicrobial activity against plant pathogens but also have antinematodal activities [38]. The combined use of modern molecular genetic methods and toolsets, enzyme engineering, as well as biocatalysts and bioinformatics, make it possible to obtain many natural plant antimicrobial compounds [39]. Genome editing (GE) tools such as the clustered regularly interspaced short palindromic repeats (CRISPR)/Cas-mediated GE have been recently used to study plant–microbe interactions for agronomic trait improvement [40]. However, understanding how plants manipulate their microbiome is important both for the design of next-generation microbial inoculants–probiotics [41] and for GE designs for targeted disease suppression and enhanced plant growth.

## 3. Farm to Fork Strategy

The European Commission’s Farm to Fork Strategy (European Commission, Eur-lex, https://eur-lex.europa.eu/legal-content/EN/TXT/?uri=CELEX:52020DC0381) (accessed on 20 May 2020), a key element of Green Europe, relates to challenges facing European agriculture and proposes measures for creating a more resilient and sustainable food system [42] based on reducing its impact on the environment while increasing its resilience and ensuring food security. Agricultural systems are complex, so “social innovation”/“technical innovation”, or any other solution, will need a tradeoff requiring public choice. For example, the goal of reducing the number of pesticides requires farmers to develop new knowledge to find other ways of controlling pests [43] and pathogenic bacteria.

## 4. Probiotics

The use of fermented milk is associated with the history of mankind, the earliest records of which date back to BC 6000 [44,45]. Even different communities in the same region, due to their cultural peculiarities (e.g., availability of raw materials of plant and animal origin), have their own unique fermented products [46]. For example, fermented products include airag (Mongolia), kefir (Russia), chhurpi (India, Nepal, Bhutan, China), dadih (Indonesia) [46], miso, shoyu, natto (Japan) [47], choratan and matsuni (Armenia) [48].

Despite the aforementioned facts, a scientific basis for the process of fermentation was discovered rather late. It was described for the first time by the founder of contemporary chemistry, A. Lavoisier, in the 1700s. Nevertheless, it was Louis Pasteur who finally proved that microorganisms are what cause lactic fermentation [49]. In 1899, Henry Tissier announced that faecal bifidobacteria are prevalent representatives of a healthy person’s gut microbiota. He offered to feed newborn babies suffering from diarrhea with bifidobacteria collected from a breastfed infant [50].

According to Krawczyk and Banaszkiewicz, the Polish doctor Józef Brudziński applied a *Bacillus lactis aërogenes* suspension in the treatment of infants with acute infectious diarrhea [51]. In 1908, a Russian microbiologist, Nobel Prize award winner Élie Metchnikoff, and Paul Ehrlich, one of the commonly acknowledged founders of probiotics [52,53,54] showed that harmful bacteria can be replaced with beneficiary ones extracted from fermented milk to treat intestinal diseases [55]. Metchnikoff linked the longevity of people’s lives with the high intake of fermented milk products [56]. Studies on probiotics were only revived by Levon Erzinkian in 1940 after Metchnikoff’s death [54]. He was the first scientist in the former USSR who recognized the probiotic era in the 1940s. Starting at the end of 1940s, L. Erzinkian, the founder of technical microbiology (biotechnology) in Armenia [52], created a new method of acidophilotherapy and provided norms for feeding acidophilic milk to babies, children and adults [52,57,58]. He isolated more than 1640 strains of acidophilic bacteria [57]. Out of them, he selected more than ten of the most active strains and used those for the treatment of bacterial dysentery at the end of 1940s and at the beginning of 1950s in infection clinics and in a military hospital in Yerevan, Armenia [58]. These strains, widely studied and used in Armenia, were unknown to the Western world until the collapse of the Soviet Union. The study of the *Lactobacillus acidophilus* n.v. ep 317/402 strain “Narine”, began in Japan in the late 1980s (https://hwpartners.co.jp/en/about-narine/ (accessed on 15 March 2014); https://www.jstage.jst.go.jp/article/milk/53/2/53_37/_article/-char/en) (accessed on 15 March 2014). In 2017, Narine expertise originating from Armenia expanded to the USA, Japan, Korea, Eastern Europe (Russia, Ukraine, Belarus), the Baltics (Latvia, Lithuania) and Central Asia (Uzbekistan, Kazakhstan) (https://hwpartners.co.jp/en/about-narine/) (accessed on 15 March 2014). Nowadays, Effective microorganisms (or EM technology) are well known in more than 140 countries around the world, developed by Professor Teruo Higa in Japan in 1982 (https://www.emrojapan.com/what/). This technology is based on more than 80 bacterial strains that are safe for humans and animals. According to the manufacturers, one of the strengths of EM is that the combination of different bacteria provides a wide range of applications for the product. EMs are used in many systems related to agriculture and environmental management (https://www.emnz.com/research/tomatos) (accessed on 15 March 2014). EM “tomato” technology is partly aimed at influencing the photosynthesis of the host, the fruit yield and the quality of the tomato plant with the help of a bacteriological vaccine [1].

The term “probiotics” as “active substances that are essential for a healthy development of life” was described by Werner Kollath in 1953 and by Ferdinand Vergin in 1954 [59,60]. Probiotics are alive bacterial cells that, when administered in adequate amounts, benefit the host’s health [61] and can have a substantial impact on the functionality of human and animal [48,62,63,64,65,66,67] organisms, as well as on plants [68]. Plant probiotics are used to increase resistance to pathogens [69] and improve yields by reducing or even eliminating chemical fertilizers [68]. There is a lot of information about the mechanisms of the action of probiotics [70,71,72,73]. A set of mechanisms that sometimes overlap relate to both the direct effects of probiotics/postbiotics and the effects of probiotics’ metabolites (Table 1).

According to Table 1, probiotics might be potential producers of water-soluble riboflavin, biotin, folate, pyridoxine and other micronutrients [48]. The results indicate that the intake of certain probiotics, probably the producers of micronutrients, is associated with a positive impact on the status of these substances (in particular, vitamin B12, calcium, folate, iron and zinc) in healthy voluntaries [76]. On the other hand, the impact of probiotics on other metabolites (hydrogen peroxide [77], lactic acid and certain short-chain fatty acids (SCFA) [74]) against the growth of competing species is also known [84]. It is also well known that the effect of probiotics can be mediated by their metabolites, such as SCFA, in particular, propionate, acetate, and butyrate, which may exercise anti-inflammatory effects [74].

Thus, the main mechanisms that determine the beneficial effects of human/animal probiotics relate to the production of antimicrobial substances; to interactions between the probiotics and the intestinal commensal and pathogenic bacteria, as well as with the host epithelium; to their impact on a pathogen’s toxin production/utilization; to immunomodulation of the innate immune response to both anti-inflammatory and pro-inflammatory directions; and to effects of probiotic bacteria on the development of the adaptive immune response (Table 1). Vertebrate immune cells provide the host with potent antigenic activity and memory; probiotic bacteria might be involved in antibody formation and development of the adaptive immune response [85,86]. Modulation of mucosal barrier function [87], as well as inhibition of neutrophil migration [88], may also be important mechanisms where probiotics can affect intestinal diseases. The direct effect of probiotics/postbiotics on gastric *Helicobacter pylori* is not excluded either [89].

Haas and Keel first used the term “Plant Probiotic Bacteria” referring to “a group of microorganisms benefiting plants, which fulfils three essential criteria that combined result in better plant protection: (i) effectiveness and competitiveness in niche colonization, (ii) the ability to create induced systemic resistance in their hosts and (iii) presence of direct antagonistic traits on pathogens” [68]. These probiotics might also be effective in alleviating stresses from salinity/heavy-metal accumulation [68]. Plants do not have specialized immune cells, although it is believed that plant cells can carry out an effective immune response based on their plant-innate immune system, including self-monitoring, system signals and chromosomal changes [90]. Plant probiotic bacteria, being promoters of vegetable food quality, also realize their beneficial role through different direct and indirect mechanisms [91]. The main supposed mechanisms of tomato probiotic bacteria are related to the biocontrol of tomato pathogens [92], phytostimulation [93,94] and nutrient mobilization [93]. Investigations on the phytostimulation effects of *Trichoderma harzianum*, *Bacillus subtilis* and arbuscular mycorrhizal fungi, through their ability to induce pathogenesis-related-proteins (chitinase, β-1,3-glucanase, peroxidase, phenylalanine ammonia-lyase as well as phenolics), in tomato plants described the effectiveness of these bacteria against *Fusarium oxysporum f.* sp. radicis-lycopersici infection [94]. Another study on approximately 400 tomato root-associated bacterial isolates (the majority belonging to *Bacillales*, *Enterobacteriales*, and *Pseudomonadales*) revealed that about 33% of these isolates produced siderophores and were able to solubilize phosphates and also revealed that about 30% of these bacteria (the majority belonging to *Bacillus* spp.) had antimicrobial activities against all the tomato pathogens tested [93]. The strains *Pseudomonas* sp. 19Fv1T [95], *Bacillus megaterium* and *Bacillus amyloliquefaciens* [96] not only are able to enhance tomato yield, but also increase the concentration of vitamin C in tomato fruits [97]. However, if *Bacillus*, *Paraburkholderia*, *Pseudomonas*, *Acinetobacter*, *Alcaligenes*, *Arthrobacter* and *Serratia* plant probiotic strains are mostly known for promoting plant growth [98], the lactobacilli probiotics might suppress several diseases in Chinese lettuce, onion, potato, and tomato [99]. At the same time, several studies have shown that the addition of probiotic lactobacilli, particularly *L. rhamnosus* strains, to human/animal/apiary food may reduce the negative effects of organophosphate pesticides used in agriculture [100].

In the modern world, it is advisable to use natural ingredients instead of chemicals and food additives not only to preserve plant biodiversity, but also to increase food storage capacity. For example, the use of probiotic strains of lactic acid bacteria (LAB)-producing bacteriocins is of great interest, since they are generally recognized as harmless microorganisms and their antimicrobial products are effective bio-preservatives. A wide variety of secondary metabolites of probiotics (pigments, vitamins, antibiotics, etc.) have important applications in human and animal health [101]. The cancer-preventive [101,102,103], antidiabetic [104,105], anti-depressive [67,106] and antihypertensive [107] effects of probiotics/their metabolites as functional ingredients have been reported previously. Moreover, the impact of probiotics on viral and respiratory tract infections has also been described [108]. It is also possible to “regulate” probiotic antagonistic activities through the use of different technologies [62]. In food technologies that use tomatoes or their processed products, it is advisable to use plant-specific probiotic strains that have previously been assessed for efficacy and safety in model food systems, as well as in adequate biological test systems [109,110]. Unlike the potential negative effects of antibiotic use [111], the use of probiotics in healthcare [112], as well as in food production, is effective in combating pathogens and has no negative consequences [113,114,115].

### “One Health” Probiotics

Soil ecosystems contain and support the largest amount of biodiversity on the planet, which mostly consists of microorganisms that are beneficial to humans and animals. The One Health concept allows us to consider some infectious diseases from three sides: harm to the environment, their impact on human health, and their impact on animal health. In general, soil and the human gut contain approximately the same number of active microorganisms [116]. However, the diversity of the human gut microbiome is only 10% of soil biodiversity [116]. Based on this knowledge and the probiotic formulation [61], “One Health” or “universal” functional probiotics were previously suggested by Malkhasyan and Pepoyan as next-generation probiotics, beneficial to both humans and their environment [117]. EM technologies mainly refer to microorganisms that are effective in soil and aquatic environments. EMs do not apply, for example, to plant and animal-origin raw material technologies or product processing. EM technologies do not oblige us to use plant probiotics for food processing (both of plant and animal origin) or for veterinary and health purposes. For example, effective microorganisms used for growing tomatoes are not ready to immediately ensure the safety of receiving and storing tomato juice, tomato paste, various sauces, canned or sun-dried fruits or powdered products. It is assumed that “One Health” probiotic microorganisms belong to 10% of microorganisms common to the human gut and soil microbiome. It is likely that, first of all, “One Health” probiotics might be the result of the screening of a new generation soil/plant/animal probiotics from “human” probiotics.

Table 2 presents the effects of lactobacilli probiotics (*Lactiplantibacillus plantarum,* L. acidophilus, Lacticaseibacillus casei and Lacticaseibacillus paracasei, Lactobacillus delbrueckii, Lacticaseibacillus rhamnosus and Leuconostoc mesenteroides) on human, animal and plant health.

LABs, the representatives of different ecosystems on Earth, exhibiting dynamic interactions within the animal and plant kingdoms in relation to other microbes, evolved along with plants, invertebrates and vertebrates, establishing either mutualism, symbiosis, commensalism or even parasitic behavior with their hosts [146]. LAB strains, also one of the main probiotic candidates [147], have been used in the production of fermented food around the world since ancient times [148]. *Lactobacillus* species, having colonizing abilities in the phyllosphere, endosphere and rhizosphere, are also able to colonize the fruits and flowers of different plants, including tomato plants [149]. Moreover, the presence of *Lpb.* *plantarum* in raw fruits indicates the fact that the plant is highly nutritious and bacteriologically healthy [145]. According to Table 2, the food-related probiotic strain *Lpb.* *plantarum* [120], showing great adaptability and adhesion in the gastrointestinal tract of host organisms, may contribute to the improvement of host gut health [120].

Studies on tomato juice containing such bacteria have shown that this product can serve as a healthy drink for vegetarians or consumers who are allergic to dairy products [122]. The beneficial effects of the *L. acidophilus* [120,128,129], *Lcb. casei* and *Lcb. paracasei* [134,135,136], *L. delbrueckii* [45,138], *Lcb. rhamnosus* [45,140] and *Leuconostoc mesenteroides* [142,143] strains on human and animal health are known as well (Table 2). As with *Lpb. plantarum*, *L. mesenteroides* determines a fruit’s “health” [145]. Furthermore, strains of *L. acidophilus* might be usable as plant growth promoting agents [131]. Regarding lactobacilli comparative viability and folate production in apple, grape and orange juice, after 48 h, viable bacterial cells are highest in fermented apple juice, which is not only the best substrate for the growth of lactobacilli, but also for the production of folic acid by *Lpb. plantarum* and *Lcb. rhamnosus* [141]. Very little is known about the effects of *L. acidophilus*, *Lcb. casei* and *Lcb. paracasei*, *L. delbrueckii* and *Lcb. rhamnosus* strains on soil or the modulation of bioaccessibility of soil heavy metals (Table 2).

Thus, *Lpb. plantarum* is a Gram-positive bacterium with a fairly large genome. It produces two isomers of lactic acid (D and L) during growth at 15 °C and 4% NaCl. Strains of *Lpb. plantarum* (and/or its bioactive products), having an ability to ferment a broad spectrum of plant carbohydrates [119], probiotic effects on human [67,150,151] and animal health [48,120,152], as well as being found in dairy products [152,153], vegetables [154], sauerkraut, pickles, some cheeses, fermented sausages, fish products [155] and rhizospheric soil [156], are probably the best candidates for “One Health” probiotics (and for “One Health—tomato” probiotics). According to Table 2, the strains of *L. acidophilus*, *L. delbrueckii*, *Lcb. casei*, *Lcb. paracasei*, *Lcb. rhamnosus* and *Leuconostoc mesenteroides* can also be considered sources of “One Health” probiotics (Table 2). It is likely to find “ready-to-use one health probiotics” in a range of probiotic strains, such as those found by Drs Erzinkian and Teruo Higa and other investigators.

Despite the vital and useful features of this bacterium, a high concentration of *Lpb. plantarum* in food can be the cause of its spoilage. It can also cause the production of mucus, sourness and green coloring even in reprocessed goods. The formation of a moderate amount of mucus is also typical of *Lactobacillus sakei* [157]. *L. lactis* is a Gram-positive bacterium used in the dairy industry, which has homofermentative metabolism and generally produces L-(+)—lactic acid [158]. Nevertheless, in cases of low pH, D-(-)—lactic acid can be produced as well. On the other hand, *L. lactis subsp. lactis*, previous *Streptococcus lactis* [159], is used in the early stages of the production of various cheese types, including Brie, Camembert, Cheddar, Colby, Gruyere, Parmesan and Roquefort [160]. A high concentration of these microorganisms infuses milk and other dairy products with apricot flavoring [161]. *Leuconostoc* spp is a Gram-positive, heterofermentative lactic acid bacterium which is capable of producing dextran out of sucrose. *Leuconostoc carnosum* was first isolated from meat kept in a refrigerator. It affects vacuumed and cooked meat by causing rotting, changes in acidity and the formation of gas and/or mucus [162]. The influence of lactobacilli on the spoilage of wine is also well known. Furthermore, this bacterium can be the reason for the decomposition of cookies, the cause of which is the heterofermentative feature of malonic acid.

## 5. Conclusions

Despite the presence of sufficient information on the processing of tomato, this data is fragmented; there is no comprehensive approach to considering the entire chain, starting from the selection of the variety and the conditions for growing raw materials, the parameters of preparing it for processing and technological methods for extracting functional ingredients. The situation is similar to the production of probiotic preparations based on cultures of commensal microorganisms for those strains that are capable of producing bacteriocins. Conducting complex scientific research in these areas is an extremely important and urgent task. On the other hand, the amount of research being conducted concerning the plant probiotics of the tomato is continuously growing. Moreover, there are investigations from ancient times that also certify the growth of both lactic acid bacteria and probiotics in tomato juice and its technological processes. Nevertheless, up until now, there have been no studies that propose the use of lactic acid bacteria from tomato fruit as stimulators of technological production.

Discussions about the “necessity” of the use of probiotics, isolated from human gut microbiota in different levels of the food chain, are also missing. It has been suggested that these probiotics should be called “One Health” probiotics. The minimal requirement for these probiotics (its concentration) is to be safe for use in different levels of the food chain and, meanwhile, to contain useful features for human health. Analysis of the literature states that lactobacilli, particularly *Lpb. plantarum* strains, can be used to ensure the biosafety of “One Health” probiotics, e.g., tomato fruit and the biotechnological processes of its production. However, upcoming developments in bioinformatics studies, based on investigations of “probiotic” genes [67,68,69,70], will contribute to the detection and use of “One-Health” probiotics from tomatoes.

## Figures and Tables

**Table 1:** Main mechanisms that determine the beneficial effects of human/animal probiotics.

Mechanism	Effect
Production of antimicrobial substances	Probiotic strains might secrete substances that may participate in the formation of endosomes [74].The effects of probiotic bacteriocins on the exhibition of selective as well as target-specific antagonistic activities in host organisms are known [75].Despite varying degrees of efficacy, the intake of certain probiotics in healthy voluntaries is associated with a positive impact on the status of certain micronutrients: vitamin B12, calcium, folate, iron and zinc [48,76]. In addition, probiotics might also be potential producers of biotin and pyridoxine [48], and the impact of lactobacilli metabolites through their chemical nature (hydrogen peroxide [77], lactic acid, folic acid, vitamins) against the growth of competing species is also known. The effect of probiotics can be mediated by their metabolites, such as short-chain fatty acid (SCFA), in particular, propionate, acetate and butyrate, which may exercise anti-inflammatory effects [74], too.
Interactions of probiotics with intestinal commensal bacteria and with pathogens	Probiotics are used to prevent/alleviate enteric infections [78]. Through the manganese transporter (MntH1), probiotics might also influence the depletion of manganese, an essential trace element, inhibiting the growth of spoilage-bacteria in dairy products [79].
Interactions of probiotics with the host epitelium	Probiotics can regulate intestinal permeability as well as compete with, for example, viruses (viral receptors) to bind receptors on epithelial cells [80].
Impact on a pathogen’s toxin production/utilization	Probiotics may have beneficial outcomes through their effect on the production of pathogen toxins [81].
Immunomodulation of the innate immune response to anti-inflammatory direction	The anti-inflammatory effects of probiotics through in vitro and ex vivo studies as well as in animal experiments is known [82].
Immunomodulation of the innate immune response to pro-inflammatory direction	Immunomodulation of the innate immune response to pro-inflammatory direction by the probiotics is known [82,83].
Adaptive immune response	Probiotic bacteria are involved in the development of adaptive immune responses [83].
Antibody formation	Probiotic bacteria are involved in antibody formation [81].

**Table 2:** Effects of lactobacilli species.

Probiotic	Human	Animal	Plant (Tomato) Products	Plant	Soil
*Lactiplantibacillus plantarum*	Food-associated *Lpb. plantarum* shows a good adaptation and adhesion ability in the gastrointestinal tract and the potential to affect host health through various beneficial activities [118].	*Lpb. plantarum* supplementation can modulate overall health and immunity [119] as well as gut microbial composition and the interaction network between gut microbiota and the immune system [120].The anti-*Helicobacter pylori* effects of the probiotic in the stomach tissue of C57BL/6 mice has also been described [89].	The use of this probiotic in the creation of fermented tomato juice products might be quite effective [113,114]. *Lpb.* *plantarum* is the main bacterial species associated with olive processing [121]. Probiotic tomato juice could serve as a health beverage for vegetarians/consumers who are allergic to dairy products [122].	This probiotic has been frequently found in environments associated with plants [123,124,125].Significant stimulation of germination in tomatoes with poor initial germination capacity was achieved by soaking their seeds for 6 h in suspensions of nine out of ten *Lpb.* *plantarum* strains tested [126].	This probiotic has highly antagonistic activities against most soil pathogens [37].
*Lactobacillus acidophilus*	The beneficial role of this probiotic in regulating imbalances in human intestinal microbiota [127], as well as in improving overall human health, is well- known [128]. The probiotic participates in the biodegradation processes [129].	*L. acidophilus* supplementation can modulate overall health, immunity, and gut microbial composition [130] as well as the interaction network between gut microbiota and animal immune system [120].	Probiotic tomato juice, containing this probiotic could serve as a health beverage for vegetarians or consumers who are allergic to dairy products [122].	The probiotic might be used as a plant growth promoting agent [131].Probiotic-loaded edible films/coatings are known for maintaining safety, quality, nutritional and functional characteristics in fruits and vegetables for longer storage periods [132].	Gut lactobacilli modulate bioaccessibility in soil lead [133].
*Lacticaseibacillus casei* and *Lacticaseibacillus paracasei*	The effects of these probiotics on skin [134] and the prevention of age-dependent cognitive decline by upregulating brain-derived neurotrophic factor expression in the hippocampus, as well as cAMP response element binding protein, were revealed in [135].	The *Lcb. casei* IMV B-7280 strain has a positive effect on the gut microbiota composition of mice [136].	Probiotic tomato juice could serve as a health beverage for vegetarians/consumers who are allergic to dairy products [122].	The effects of probiotic-loaded edible films/coatings on the maintainance of safety, quality and nutritional and functional characteristics of fruits and vegetables for long storage periods are known [132]. Furthermore, the effect of gamma-irradiated probiotics as an edible coating to enhance the storage of tomato under cold storage conditions is known [137].	
*Lactobacillus delbrueckii*	The effects of this probiotic on immune responses in the elderly were described in [138].	The efficiency of this probiotic’s applications on pre- and post-radiation nutrition for rats were described in [48].	Probiotic tomato juice could serve as a health beverage for vegetarians or consumers who are allergic to dairy products [122].	Plant-originated *L. bulgaricus* was described by Michaylova and coauthors [139].	
*Lacticaseibacillus rhamnosus*	The effects of this probiotic on the modulation gut microbiota were described in [140].	The efficiency of this probiotic on pre- and post-radiation nutrition for rats were described in [48].	Fermented apple juice was the best substrate for the production of folic acid via *Lpb. plantarum* and *Lcb. rhamnosus* [141].	The effects of probiotic-loaded edible films/coatings on the maintainance of the safety, quality and nutritional and functional characteristics of fruit and vegetables for long storage periods are known [132].	
*Leuconostoc* *mesenteroides*	The effects of this probiotic on the age-related decline in T cell-related immune functions were shown in [142].	This bacteria has great potential as a bee probiotic and could enhance the health of bee colonies [143].	This bacteria, being one of the predominant in the tomato surface microbiome, helps to control contaminate proliferation on tomato purée during storage at abusive temperatures [144].	The presence of this bacteria in raw fruits indicates the fact that the fruit is highly nutritionally and bacteriologically healthy [145].	
